# Supplementary material for: Effect of Intragastric Botulinum Type A Injection Combined with a Low-Calorie High-Protein Diet in Adults with Overweight or Obesity
Source: J Clin Med. 2022 Jun 10;11(12):3325. doi: 10.3390/jcm11123325 (PMC9225463; doi:10.3390/jcm11123325)
Supplement: Supplementary file 1 [file jcm-11-03325-s001.zip › jcm-1753809-supplementary.pdf]

Supplementary Figure S1. Endoscopic injection of botulinum toxin in the antrum of stomach

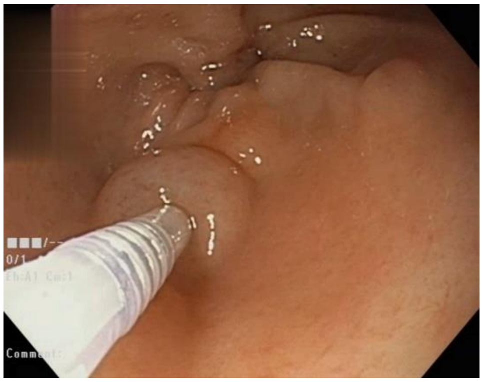

Method:

Inject 30ml of botulinum type A (Botox; Allergan, Irvine, CA) containing 300 units in 0.9% normal saline through a 25-gauge needle, and inject 10ml each on antrum, body, and fundus through submucosal injection.

Supplementary Table S1 – Multivariate logistic regression analysis of factors associated with weight loss more than 5% in IGBI group, adjusted for sex and age

**Variable**	**IGBI**	
	**Adjusted OR (95%CI)**	**P-value**
Weight (kg)	1.0(0.95-1.10)	0.431
BMI (kg/m^2)	0.9(0.69-1.22)	0.588
HBA1c (%)	0.6(0.11-3.55)	0.604
Cholesterol (mg/dL)		
Total	1.0(0.96-1.17)	0.237
HDL cholesterol	0.9(0.82-1.01)	0.089
LDL cholesterol	0.9(0.86-1.05)	0.345
Triglycerides (mg/dL)	0.9(0.98-1.01)	0.550

OR: Odds ratio; 95%CI: 95% confidence interval; IGBI: Intragastric botulinum injection
